# Supplementary material for: Understanding the structure of cognitive noise
Source: PLoS Comput Biol. 2022 Aug 17;18(8):e1010312. doi: 10.1371/journal.pcbi.1010312 (PMC9423631; doi:10.1371/journal.pcbi.1010312)
Supplement: S1 Supporting information — Text A: Data Analysis Methods. Text B: Sampling Algorithms. Text C: Model Comparison Methods. Table A: Model scores for the three sampling algorithms in the animal naming experiment using the alternative method for generating IRIs. (PDF) [file pcbi.1010312.s001.pdf]

Supporting Information

Text A. Data Analysis Methods

Animal Naming Preprocessing and Descriptives

Submissions of animal names were pre-processed by applying spelling corrections, removing punctuation and whitespace characters, removing capitalization and pluralization, and were finally normalized by selecting unified animal names (for instance, "meal worm" as "worm" or "chimp" as "chimpanzee"). The normalization adhered to the animal taxonomy in [1] whenever possible. After normalization, a total of 411 unique animal names remained. Participants submitted an average of 148.2 unique animal names ($SD = 41.8$). To assess which categories subsequently named animals belonged to, we applied the category labels used in [1, 2]. Only 79 unique animals in our experiment were not covered by these category labels (139 submissions in total). The first two authors then individually supplied category labels for these remaining animals and devised a categorization scheme consistent with the labels in [1, 2]. After applying these labels, 18 unique animals without a category label remained (48 submissions in total).

We then analyzed the proportion of within-class and between-class transitions for each participant. Transitions were scored as within-class if at least one of the category labels for subsequent animals matched the categories of the previous animal. If no category label existed for an animal, that transition was always scored as between-class. On average, 46.45% transitions were within class. This proportion was significantly larger than chance ($p < .001$, obtained via 10,000 bootstrapped samples). Transition proportions for individual participants ranged from 34.63% - 56.31% ($M = 46.87\%, SD = 6.38\%$), whereas proportions in bootstrapped independent samples were significantly lower (all $p < .001$,M=14.82%, SD=5.2%). Finally, the duration between successive animals was significantly longer ($t(18) = 2.18, p = .04$) for between-class transitions ($M = 6.94s, SD = 14.4$) than within-class transitions ($M = 3.81s, SD = 6.11$).

To assess whether IRIs correlated with distance in a semantic space, we used the semantic embedding from [1] obtained via the BEAGLE model of [3] trained on Wikipedia. We then transformed the cosine similarities between successive animals x and y into Euclidean distances via $d(x, y)^2 = 2 - 2 * sim(x, y)$. Animals for which no corresponding embeddings were available in [1] were dropped from the analysis (139 out of 4967 submissions, 2.8%). There was a positive correlation between IRIs and semantic distances, $r = 0.14, n = 4697, p < .001$. For individual participants we obtained correlations between 0.04 and 0.25 ($M = 0.15, SD = 0.07$, 8 out of 10 $p < .05$). Participants sometimes repeated the previous animal names (min = 0.38% repetitions, max = 10.70% $M = 3.63\%, SD = 2.95\%$).

Data Fitting Methods

Correlation and tail exponents of the time series from both experiments were fitted following conventional methods [4, 5]. For the correlation exponents (α), we conducted a Fourier analysis using a periodogram function in MATLAB 2020b. The correlation exponent was computed as the negative slope of the fitted line in the windowed, log-binned power spectrum for frequencies less than 0.1, following [5].

For the tail exponents (μ), we first calculated the distribution of successive changes. The changes are inter-response-intervals and absolute differences between two time estimates in the animal naming experiment and in the time estimation experiment respectively. These distributions were then fitted by a power-law distribution with the exponent and the range of data as free parameters [4]. The range was calculated as the part of the dataset with the lowest Kolmogorov-Smirnov (KS) statistic. This was implemented by repeatedly increasing the lower bound and decreasing the upper bound until the KS statistic failed to improve consecutively five times [4]. The power-law exponent was then fitted to this subset of the data using maximum-likelihood estimation [6]. In the animal naming experiment, the median fitted range was [2.00s, 61.64s]. In the time estimation experiment, the median fitted ranges were [0.02s, 1.05s], [0.03s, 2.32s], and [0.01s, 4.10s] for 1/3s, 1s, and 3s conditions respectively. Using ranges in this way helped exclude resting periods and lapses of attention from the analysis.

In a separate analysis, we looked for a signature of rest periods or lapses of attention in the time estimation experiment: changes in the time estimate that were followed by an immediate reversion to the previous time estimate. In these cases, the ratio of two consecutive changes would be close to -1. To check whether the apparent heavy tails were due instead to resting periods or inattention, as a conservative test we excluded changes in time estimates associated with change ratios between -1.2 and -0.8, and reanalyzed the remaining data. Per-participant tail exponents ranged from 0.87 to 2.45 ($M = 1.56, SD = 0.56$), and 24 of 30 were classified as heavy-tailed.

Whether a distribution follows a power law or not is often controversial and technically challenging to establish. To strengthen our results further, we also fitted our data with an alternative, strict approach. As before, we estimated power-law statistics by minimizing the KS-distance of our observed data and the theoretical MLE distribution, as suggested in [6]. Instead of the iterative procedure of determining lower- and upper bounds, we fitted a truncated power-law distribution (a power-law distribution times an exponential distribution) with a x_{min} truncation. We then contrasted the optimized power-law distribution with the fit of an exponential distribution and report normalized likelihood ratios, as well as bootstrapped p-values [6] using the implementation in [7]. Using this strict test, for all three tapping targets we obtain μ exponents suggesting power-law distributions ($\mu_{1/3s} = 1, \mu_{1s} = 1.37, \mu_{3s} = 1.82$). For both 1s and 3s targets, the likelihood ratio strongly and significantly favored the power-law distribution over an exponential ($\text{llr}_{1s} = 2.04, p_{1s} = 0.04$, $\text{llr}_{3s} = 14.87, p_{3s} < .001$). However, for the aggregate 1/3s targets the likelihood ratio was not conclusive ($\text{llr}_{1/3s} = -.04, p_{1/3s} = .97$). For aggregate animal-naming data, we found that the likelihood ratio strongly and significantly favored a power-law distribution ($\mu = 1.91$, llr = 6.91, $p < .001$).

Individual participants' μ estimates for the 1/3s target ranged from 1 to 3.9 ($M = 2.64, SD = 1.24$) and eight likelihood ratios favored power-law distributions over exponential, but most ratios were not significant (only 3/10 $p < .05$). For the 1s target, individual participants' estimates ranged from 1 to 3.23 ($M = 1.97, SD = 0.78$) and again eight likelihood ratios favored power-law, but only two likelihood ratios were significant (2/10 $p < .05$). Finally, for the 3s target, estimates ranged from 1 to 2.9 ($M = 1.58, SD = 0.69$), and nine likelihood ratios favored power-law.

In contrast to the 1/3 and 1s targets, the majority of likelihood ratios were significant (6/10 $p < .05$). For the animal naming data, estimates ranged from 1 to 2.59 ($M = 1.51, SD = 0.53$) and all likelihood ratios favored power-law over exponential, with six ratios significantly favoring the power-law (6/10 $p < .05$). Overall, using this strict test, we continued to find evidence for power-law tails in trial-by-trial changes, though in a smaller proportion of participants.

Validating the Data Fitting Methods

The identification of $1/f$ noise and heavy tails depends on the fitting methods. While the methods we used are established in the literature [5, 6, 4] and a comprehensive evaluation of the methods is beyond the scope of this paper, it is useful to test the fitting methods on a simple counterexample to demonstrate their validity. In particular, we drew independent and identically distributed (i.i.d.) samples from a standard Gaussian distribution from which the simulated traces should never exhibit $1/f$ noise or heavy tails. Traces of 2^{11} i.i.d. samples from a Gaussian were repeatedly simulated 1000 times and were fitted using the same methods as used with the data and simulations from the sampling algorithms. We found that the range of correlation exponents for these simulated traces was [-0.4989, 0.2592], clearly outside the range of $1/f$ noise [0.5, 1.5]; and that the range of tail exponents was [4.0506, 17.7245], again outside the range of heavy tails (1,3] – thus none of the 1000 replications met the criterion for $1/f$ noise or the criterion for heavy tails. This practice shows that the fitting methods are robust against a simple counterexample: i.i.d. sampling from a Gaussian.

We also show that the fitting method is robust using randomly reshuffled cognitive time series. For the animal naming task, the reshuffled time series had a 0.06% chance of being classified as $1/f$ noise across 1000 reshuffles. For the time estimation task, 0% of the reshuffled time series was classified as $1/f$ noise. These results argue against $1/f$ noise occurring by chance for values independently drawn from a heavy-tailed distribution.

Text B. Sampling Algorithms

The three sampling algorithms discussed in the main text are forms of Markov Chain Monte Carlo (MCMC). While there are elaborate versions of these algorithms with sophisticated tuning schemes that improve their efficiency, we compared simple commonly-used implementations of each.

Random-Walk Metropolis

Random Walk Metropolis (RWM; see [8] for an introduction) is a basic and well-known sampling method for sampling from a target distribution $\pi(x)$. It was run with a normal proposal distribution centered on the current state of the sampler (i.e., with zero mean and standard deviation σ).

Hamiltonian Monte Carlo

Hamiltonian Monte Carlo (HMC; see [8] for a conceptual introduction) is an MCMC algorithm that uses Hamiltonian dynamics to make better proposals. We ran HMC with the common leapfrog integration scheme and Gaussian momentum proposals. For pseudocode of the algorithm,

Algorithm 1 Random-Walk Metropolis

```
Given σ, iterations
Initialize x_0
for i = 1 : iterations do
    x_i ← MCMC STEP(x_{i−1}, π, σ)
end for
function MCMC STEP(x, π, σ)
    x' ← x + N(0, σ)                    ▷ Propose a move from the current state
    A ← min{1, π(x')/π(x)}
    if A > u ~ U_[0,1] then
        x ← x'
    end if
    return x
end function
```

see the following pseudo-code, where ϵ is the step-size parameter of the integrator, $\mathcal{L}$ is the log-likelihood of variables x, and L is the number of leapfrog integration steps.

Algorithm 2 HMC

```
Given ϵ, L, 𝓛, iterations
Initialize x_0
for i = 1 : iterations do
    r' ~ N(0, I)                                  ▷ Propose new momentum
    for l = 1 : L do
        x' ← LEAPFROG(x, r', ϵ)
    end for
    A ← min{1, exp(𝓛(x') − ½ r'·r') / exp(𝓛(x) − ½ r·r)}
    if A > u ~ U_[0,1] then
        x ← x'; r ← r'
    end if
end for
function LEAPFROG(x, r, σ)                        ▷ Leapfrog integrator
    r' ← r + (σ/2)∇_x 𝓛(x)
    x' ← x + ϵr'
    r' ← r' + (σ/2)∇_x 𝓛(x')
    return x,' r'
end function
```

Metropolis-coupled Markov Chain Monte Carlo

Metropolis-coupled Markov Chain Monte Carlo (MC^3; see [9] for an introduction) is a more sophisticated sampling algorithm that consists of multiple MCMC chains running at different temperatures. We ran several chains in parallel, with the first chain exploring the untempered distribution, and each successive chain exploring an increasingly tempered distribution. While sophisticated temperature schemes have been suggested in the literature, we adopt a linearly spaced temperature scheme, where successive chains explore increasingly tempered distributions.

Algorithm 3 Metropolis-coupled Markov Chain Monte Carlo

Given $\pi, M\text{chains}, iterations, \textsc{Temperature}, \sigma$
$T_c \leftarrow \textsc{Temperature}(c)$, for all chains c ▷ Initialize temperature
Initialize $x^c_{i=0}$, for each chain c in C chains ▷ Set the initial state of each chain
for $i = 1$: iterations **do**
  for $c = 0 : C$ **do**
    $x^c_i \leftarrow \text{MCMC } \textsc{Step}(x^c_{i-1}, \pi^{1/T_c}, \sigma)$ ▷ Perform one MCMC step for the tempered distribution π
  end for
  for $s = 0 : M/2$ **do**
    Select two chains, k, l, at random ($k \neq l$).
    $A \leftarrow \min\{1, \frac{\pi(x^k_i)^{1/T_l} \times \pi(x^l_i)^{1/T_k}}{\pi(x^k_i)^{1/T_k} \times \pi(x^l_i)^{1/T_l}}\}$ ▷ Metropolis-coupled swapping rule
    if $A > u \sim \mathcal{U}_{[0,1]}$ **then**
      $x^k_i \leftarrow x^l_i$; $x^l_i \leftarrow x^k_i$ ▷ Swap the two chains
    end if
  end for
end for

Text C. Model Comparison Methods

As noted in the main text, we conducted simulation-based model comparisons for the three sampling algorithms using the Approximate Bayesian Computation (ABC) method [10]. Sampling algorithms were simulated to produce a time series that were then compared to the observed cognitive time series. The contrast between simulated and observed time series is done through the summary statistics of both time series; in our case, we focus on the correlation (α) and tail indices (μ) as the summary statistics.

More formally, model performances were measured by the marginal likelihood, which can be approximated as follows:

$$p(D) = \int d\theta p(D|\theta)p(\theta) \tag{1}$$

$$\approx \frac{1}{N}\sum_{i=1}^{N} p(D|\theta_i), \quad \text{where} \quad \theta_i \sim p(\theta) \tag{2}$$

$$\approx \frac{1}{N}\sum_{i=1}^{N} \rho(\hat{D}(\theta_i), D), \quad \text{where} \quad \theta_i \sim p(\theta) \tag{3}$$

where θ is the set of free parameters of algorithms, N is the number of simulated time series, $\hat{D}(\theta_i)$ is the simulated time series given model parameters θ_i, and $\rho(\hat{D}, D)$ is the likelihood approximation function that compares the summary statistics between simulated and observed time series. To avoid strong preferences in the parameter space, all parameters for all three samplers were uniformly distributed across a predefined space (detailed below). For each task, each sampler was repeatedly simulated 1000 times with randomized parameter values. Both MC^3 and RWM were simulated for 2^{11} iterations. Since HMC is more sample efficient, but also computationally more costly, we ran HMC simulations for 2^8 iterations. To make the summary statistics more comparable, each simulated time series also used the same initial value.

The likelihood approximation function $\rho(\hat{D}, D)$ was computed based on the difference in correlation and tail indices between simulated and actual data. These differences were smoothed through a similarity kernel function to return a probability of the observed data given the model simulation. We assumed Gaussian kernels with mean equal to that of the participant's index and standard deviation equal to that of the cross-participant indices from the same experimental condition. Covariances of the Gaussian kernels were set to zero. The calculation of correlation and tail exponents of the simulated time series followed the same methods used in the observed data [6, 5] except that no exclusion was applied on the simulated time series.

Target Distributions

We used two target distributions to represent the hypothesis spaces of time estimates and animal names. For the time estimation experiment, a standard Gaussian distribution was assumed to be the target. For the animal naming experiment, a more sophisticated target distribution was considered. We first embedded all unique animal names that were reported by participants into a 2-dimensional space using multidimensional scaling [11], trained on the similarities in [1]. To infer a probability distribution of this semantic space, we then fitted a Dirichlet Process Gaussian Mixture Model via variational inference (with a maximum of 10 components), as implemented in [12]. Dropping mixtures with weights that were less than 0.1, we obtained a mixture of four Multivariate Gaussian distributions, for an illustration see Fig 2.

Parameter Ranges

Parameters were randomly drawn from sampler-specific priors. For RWM and MC^3, we varied the proposal width in relation to the width of the target distribution $\sigma_{prop}/\sigma_{tar}$. We employed a two-step sampling procedure, first sampling if the proposal width is larger or smaller than the target width with equal probability. Then we sampled x uniformly $x \sim \mathcal{U}(1, 10)$, resulting in samples from the range $[\sigma_{tar}/x, x/\sigma_{tar}]$, including both proposal distributions that were significantly larger than the target distribution and those that were much narrower than the target distribution. This was important because the proposal width directly affects the samples' autocorrelation: very wide proposal distributions will result in proposals outside of the typical set of the target distribution, producing large numbers of rejections, and thus, since the current state is rarely updated, large autocorrelations. However, this behavior can be subtly different for target distributions that are multimodal or for heavy-tailed distributions. In these cases, occasional large jumps might occur when a proposal into the tails or the other mode is proposed.

In addition to the proposal width, for MC^3, we sampled the number of parallel chains, M, to be between 2 and 10 chains, with equal probability. We did not vary the temperature function, and all simulations used a linearly increasing temperature between chains (increasing temperature by 5 in each chain).

For HMC, we kept the step size constant at 0.1, as it only affects the integration accuracy. We varied the path length, sampling uniformly $L = \mathcal{U}(1, 5)$. All three sampling algorithms were initialized at zero.

Generating IRIs from Samples

For the analysis in the main text, we assumed that each sample took additional time, and that the time between subsequent animal names was related to the number of samples generated before there was a change in the nearest animal name to the state of the sampler in the semantic space. With this method, the time taken to generate a sample was unrelated to the distance it had travelled, and instead followed a Poisson process: the time between each sample was exponentially distributed with rate parameter 0.1. These assumptions about the time required to generate samples match those in the Autocorrelated Bayesian Sampler model [13].

An Alternative Method for Generating IRIs

As a robustness check for the animal naming task, an alternative cognitive mechanism for IRI was considered where the IRIs are related to the Euclidean distances between two successive animal names. That is, we calculated the distance the sampler travelled in the semantic space before the nearest animal name in the space changed. We performed the same approximate Bayesian computation to evaluate model performances and found that MC^3 and RWM were worse than HMC (see SI Table 1). However, comparing to log marginal likelihood results in Table 1, HMC was outperformed by both RWM and MC^3 when IRI was related to number of samples until a new animal was nearest.

Table A. Model scores for the three sampling algorithms in the animal naming experiment using the alternative method for generating IRIs.

Model Scores	RWM	HMC	MC^3
log marginal likelihood	-109.3603	-20.0879	-103.6791
number of best-fitting participants	0	10	0
protected exceedance probability	0.0008	0.9983	0.0009

References

[1] Hills TT, Jones MN, Todd PM. Optimal foraging in semantic memory. Psychological Review. 2012;119(2):431.

[2] Troyer AK, Moscovitch M, Winocur G. Clustering and switching as two components of verbal fluency: evidence from younger and older healthy adults. Neuropsychology. 1997;11(1):138.

[3] Jones MN, Mewhort DJ. Representing word meaning and order information in a composite holographic lexicon. Psychological Review. 2007;114(1):1.

[4] Humphries NE, Weimerskirch H, Queiroz N, Southall EJ, Sims DW Foraging success of biological Lévy flights recorded in situ. Proceedings of the National Academy of Sciences. 2012;109(19):7169–7174.

[5] Gilden DL, Thornton T, Mallon MW. $1/f$ noise in human cognition. Science. 1995; 267(5205):1837–1839.

[6] Clauset A, Shalizi CR, Newman MEJ Power-law distributions in empirical data. SIAM review. 2009;51(4):661–703.

[7] Alstott J, Bullmore DP. powerlaw: a Python package for analysis of heavy-tailed distributions. PloS One. 2014;9(1).

[8] MacKay DJ. Information theory, inference and learning algorithms. Cambridge University Press; 2003.

[9] Altekar G, Dwarkadas S, Huelsenbeck JP, Ronquist F. Parallel metropolis coupled Markov chain Monte Carlo for Bayesian phylogenetic inference. Bioinformatics. 2004;20(3):407–415.

[10] Turner BM, Van Zandt T. Approximating Bayesian inference through model simulation. Trends in cognitive sciences. 2018;22(9):826–840.

[11] Borg I, Groenen PJ. Modern multidimensional scaling: Theory and applications. Springer Science & Business Media; 2005.

[12] Pedregosa F, Varoquaux G, Gramfort A, Michel V, Thirion B, Grisel O, et al. Scikit-learn: Machine Learning in Python. Journal of Machine Learning Research. 2011;12:2825–2830.

[13] Zhu JQ, Sundh, J, Spicer, J, Chater, N, Sanborn, AN. The Autocorrelated Bayesian Sampler: A Rational Process for Probability Judgments, Estimates, Confidence Intervals, Choices, Confidence Judgments, and Response Times. PsyArXiv. 2021.
