# Supplementary material for: Understanding the structure of cognitive noise
Source: PLoS Comput Biol. 2022 Aug 17;18(8):e1010312. doi: 10.1371/journal.pcbi.1010312 (PMC9423631; doi:10.1371/journal.pcbi.1010312)
Supplement: Attachment — Submitted filename: response_letter.pdf [file pcbi.1010312.s002.pdf]

Dear Dr. Gershman,

We would like to thank the Reviewers for their careful reading of our manuscript and for their very valuable comments. The changes we have made in response have greatly strengthened the paper. In particular, our biggest change was conducting sophisticated fits to the psychological data to establish which sampling algorithm best fit the data, finding stronger support for the MC^3 algorithm than in our original submission.

Below we describe how each of the points raised has been addressed in the revision. Our responses are in blue and the parts of the manuscript that has been modified are in red.

Kind Regards,

Zhu, Pablo, Adam, and Nick

Dear Dr. Zhu,

Thank you very much for submitting your manuscript "Understanding the Structure of Cognitive Noise" for consideration at PLOS Computational Biology.

As with all papers reviewed by the journal, your manuscript was reviewed by members of the editorial board and by several independent reviewers. In light of the reviews (below this email), we would like to invite the resubmission of a significantly-revised version that takes into account the reviewers' comments. Reviewers 1 and 3 are relatively positive, but Reviewer 2 recommends rejection. I find Reviewer 2's arguments fairly compelling, but I also think that you might be able to address them. In addition, I agree with Reviewer 1 that 27% (while greater than 4% and 2%) is not particularly impressive in absolute terms. I'm not sure that this result provides such compelling evidence for MC3.

We cannot make any decision about publication until we have seen the revised manuscript and your response to the reviewers' comments. Your revised manuscript is also likely to be sent to reviewers for further evaluation.

When you are ready to resubmit, please upload the following:

[1] A letter containing a detailed list of your responses to the review comments and a description of the changes you have made in the manuscript. Please note while forming your response, if your article is accepted, you may have the opportunity to make the peer review history publicly available. The record will include editor decision letters (with reviews) and your responses to reviewer comments. If eligible, we will contact you to opt in or out.

[2] Two versions of the revised manuscript: one with either highlights or tracked changes denoting where the text has been changed; the other a clean version (uploaded as the manuscript file).

Important additional instructions are given below your reviewer comments.

Please prepare and submit your revised manuscript within 60 days. If you anticipate any delay, please let us know the expected resubmission date by replying to this email. Please note that revised manuscripts received after the 60-day due date may require evaluation and peer review similar to newly submitted manuscripts.

Thank you again for your submission. We hope that our editorial process has been constructive so far, and we welcome your feedback at any time. Please don't hesitate to contact us if you have any questions or comments.

Sincerely,

Samuel J. Gershman

Deputy Editor

PLOS Computational Biology

****

Reviewer's Responses to Questions

Comments to the Authors:

Reviewer #1: This paper explores two interesting features of human response-time distributions: (a) their heavy-tailed shape and (b) their autocorrelation over long time periods (1/f noise). Although there have been theories that explain each of these features of RTs independently, the authors show that they can co-occur in two disparate cognitive tasks (animal naming and rhythmic time estimation), which suggests a potential common explanation for (a) and (b). In simulations, the authors show that both of these features can arise from a specific type of MCMC algorithm (MC3), which efficiently transitions between different chains when one chain seems to find a novel mode. Thus, if the human brain is implementing a similar algorithm when performing tasks like animal naming, as some cognitive theories suggest, then this provides a parsimonious explanation for both the heavy-tailed RT distribution and 1/f noise.

I enjoyed reading this paper and found the ideas intriguing. Although I believe the work is well-worth being out in the literature, it would be helpful for the authors to fill in some key details in a revision.

We thank the Reviewer for these kind comments.

Perhaps this is partly explained by my relative unfamiliarity with the subject matter, but I suspect many people would benefit if the authors put in more effort to connect the empirical section of the paper with the simulation work. For example, as of now, I don't even have a sense of exactly how the authors envision that the time estimation task might use an MCMC algorithm. The task involves rhythmically tapping the space bar at the same tempo for a long period of time. This doesn't strike me as the kind of cognitively taxing prediction/estimation task that MCMC is typically used for. Moreover, although I have some sense of how MCMC could be relevant for the animal naming task, I'm still not quite sure how to map the MCMC algorithm onto the behavior. For example, when an animal pops into a person's mind, what is the corresponding 'event' in MCMC? Presumably subjects would not be reporting literally every sample from that algorithm, so what constitutes a sample that reaches conscious awareness?

Thank you for this suggestion. Most broadly, we believe that the time estimation task involves cognition because the character of the responses depends on the predictability of the sequence: Gilden, Thornton, and Mallon (1995) found that if people were instructed to respond at unpredictable intervals, the time series of response times appeared to show sequential independence like white noise, in contrast to the sequential dependencies (i.e., 1/f noise) found if instructed to respond at regular intervals. More specifically, there are existing Bayesian models of time estimation, which we now cite in the main text (Ahrens & Sahani, 2011; Roach et al., 2017). We are not aware of MCMC algorithms having been applied to time estimation tasks in past work, but they have been applied to other perceptual tasks such as bistable perception (Gershman, Vul, & Tenenbaum, 2012), and numerosity estimation (Spicer et al., in press).

For the time estimation task, we assume that participants report every sample that is produced using a sampling algorithm and time estimates are direct readouts of the samples. For the animal naming task, we assume that participants are aware of the closest animal name in the semantic space to the current position of the sampler. We further hypothesize that the inter-response-interval between the two successive animal names are on average proportional to the number of samples generated before the nearest animal name in semantic space changes. Samples are assumed to be generated at a fixed rate but at random points in time, following other modeling work on decision response times (Zhu et al., 2021). We use the change in animal name to mark a new report, which corresponds to the instructions we gave participants. We now explicitly describe our behavioral model for converting samples to behavior in the main text and in more detail in the Supplementary Text.

References:

Gilden, D. L., Thornton, T., & Mallon, M. W. (1995). 1/f noise in human cognition. *Science*, *267*(5205), 1837-1839.

Ahrens, M. B., & Sahani, M. (2011). Observers exploit stochastic models of sensory change to help judge the passage of time. *Current Biology*, *21*(3), 200-206.

Roach, N. W., McGraw, P. V., Whitaker, D. J., & Heron, J. (2017). Generalization of prior information for rapid Bayesian time estimation. *Proceedings of the National Academy of Sciences*, *114*(2), 412-417.

Gershman, S. J., Vul, E., & Tenenbaum, J. B. (2012). Multistability and perceptual inference. *Neural computation*, *24*(1), 1-24.

Spicer, J., Zhu, J.-Q., Chater, N., & Sanborn, A. N. (in press). Perceptual and Cognitive Judgments Show Both Anchoring and Repulsion. *Psychological Science*.

Zhu, J.-Q., Sundh, J., Chater, N., & Sanborn, A. N. (2021). The Autocorrelated Bayesian Sampler for Estimation, Choice, Confidence, and Response Times. https://doi.org/10.31234/osf.io/3qxf7

I also worry a bit about the generalizability of the simulation results. Even for the MC3 algorithm, only 27% of parameterizations yielded the combined distributional features that are characteristic of human responses. Is there good reason to believe that these parameterizations — or the MC3 algorithm itself, for that matter — are psychologically plausible? This may be a difficult question to answer given the lack of research on this topic, but more context for how these parameters relate to psychology would be useful for assessing the plausibility of the theory. Furthermore, why was the MCMC algorithm only run on relatively simple probability distributions? Why not model an environment that resembles the more patchy semantic network for animals or other task-relevant domains?

Thanks for these suggestions – our response to them and to similar comments from the other reviewers and the editor constitute the bulk of the work we did for this revision. The evaluation of the proportion of parameterizations that yield the combined distributional features has now been removed from the paper, as it did not address which parameters best fit the human data. Instead, our main analysis is now a more standard model comparison, using the method of Approximate Bayesian Computation, that puts the three samping algorithms in direct competition in terms of how well they fit (corrected for model complexity) the distributional features of individual participants. We calculated both an overall goodness of fit for each algorithm as well as the proportion of participants best fit by each algorithm. Along both measures, MC3 comprehensively won these comparisons for the time estimation task data and was a close second to the best algorithm (RWM) for the animal naming task data.

Aside from assessing which model is relatively the best, we also assessed whether MC3 was able to produce heavy tails and 1/f noise using the best-fitting parameters. Using the posterior predictive mode of the parameters, we found that MC3 produced both features very often, corresponding perfectly with participants in the animal naming task and corresponding strongly with participants in the time estimation task.

In this model comparison, we took onboard the comment that the representations we used were not task-specific. The model comparison for both tasks now is based on more realistic representations. The time estimation task uses a Gaussian distribution, as that seemed to at least roughly match the distribution of human responses. The animal naming task now uses a patchy 2D semantic space constructed from fitting a Gaussian mixture model to the animal name responses. The relationship between the generated samples and the outcome of interest (i.e., IRIs and time estimates) have been updated as detailed in our response to the preceding comment.

In sum, the theoretical ideas explored in this paper seem quite interesting, but the authors could expand upon the ways in which their simulation models connect to the psychological phenomena of interest and provide more detail about how well the models really capture human behavior.

Minor:

- I’m not sure what this means: “1/f noise has been explained as the result of complex organisms displaying a self-organized criticality” (p. 4).

We replaced the sentence with “1/f noise has been explained as the result of complex organisms displaying a self-organized criticality, meaning the organisms are inherently and continuously transitioning between different stable states and as a consequence exhibit complex behavior (Van Orden, Holden, & Turvey, 2003)”

- In Figure 1 (or another figure), it would be helpful to see some simple histograms of participants' actual RTs. I don't have a great sense of how well these distributions are fitted by a power law or exhibit 1/f autocorrelation. Is there any way to directly overlay model predictions on data?

To illustrate the individual-level fit, we have now plotted both the histograms and the power spectral density in conjunction with the fitted curves and lines for each participant. These plots are now shown in the Supplementary. (The histograms of actual RTs are able to illustrate heavy-tail changes, but are not able to show 1/f autocorrelations. This is because the 1/f autocorrelations depend on the particular ordering of the trials, as illustrated in our response to Reviewer 3's comment on how often 1/f noise appears when trials are reshuffled.)

Reviewer #2: The authors show that two human tasks exhibit both heavy tailed distributions of trial-to-trial differences as well as 1/f autocorrelations. They posit that the co-occurrence of these two statistical properties is difficult to explain under standard accounts, and thus might reveal something about the cognitive mechanisms underlying the noise. Finally, they argue that Metropolis-coupled MCMC exhibits the same combination of heavy-tailed, 1/f autocorrelated behavior, suggesting that this is what people are doing. I have two major reservations about this paper: (1) I am not convinced that the combination of heavy-tailed distributions and 1/f autocorrelations is particularly exotic, and (2) i am not convinced that the characterization of MC^3 dynamics is sufficiently diagnostic. Together, these two reservations undermine the major contribution of this paper: the suggestion that the correspondence of these dynamics in human behavior and inference algorithms reveals something about human cognitive mechanisms.

We thank the Reviewer for the critical evaluation of our manuscript. As elaborated in detail below, we wish to stress that (i) the co-occurrence of heavy tails and 1/f noise may seem ubiquitous in nature but is still novel in human cognitive behavior and so deserves an explanation in this context; (ii) we now better explain the diagnosticity of the fitting process and, because the simulation evidence we presented in the original submission was not satisfactory, we now present a model comparison as our main analysis.

How rare is the co-occurrence of heavy-tailed difference distributions and 1/f autocorrelations? This is the first time I have heard the claim that the co-occurrence is rare, so I went to the obvious place to check: natural images. I downloaded a random grayscale natural image from google image search, pulled out one row of pixels, and evaluated the heavy-tail-ness of the pairwise pixel luminance difference, as well as the log-log slope of the power spectrum. I got both -- the log-log slope of the power spectrum was -0.6, and the log-log slope of the mass in the tails of the distribution was -1.3. So, the first place I looked that has natural 1/f noise also has heavy tails for the adjacent pairwise distances (according to the authors' criteria). If luminance in natural images has this co-occurrence as well, I am not sure what we have to learn about cognitive mechanisms from this co-occurrence. If the authors want to make any kind of argument about cognitive mechanisms from these characteristics of response time distributions, it seems important to show that heavy tailed differences are rare among the many natural phenomena that exhibit 1/f noise (https://en.wikipedia.org/wiki/Pink_noise#Occurrence), because if DNA sequences, quasar light emissions, meteorological data series, are like luminance in natural images and response times, and also exhibit the conjunction of 1/f noise and heavy tailed distributions, then appealing to a particular cognitive mechanism to explain the co-occurrence sounds a bit far-fetched.

The Reviewer is making an important point that we could have explained more clearly the distinction in the initial submission. As the demonstration of the Reviewer showed, the co-occurrence of heavy tails and 1/f noise is repeatedly demonstrated in natural statistics. However, we highlight that such co-occurrence was rarely discussed in the cognitive science literature.

In particular, the co-occurrence is entirely absent in conventional cognitive models. Most cognitive models assume i.i.d. noise, in an apparent contradiction with the finding of 1/f noise. The few exceptions that we are aware of include

the Wagenmakers, Farrell, & Ratcliff's (2004) drift-diffusion model whose drift rate parameter follows an autoregressive process. However, this model is limited to predict binary choices, and so does not apply to either our animal naming or time estimation tasks. In addition, heavy tails in successive changes have received very little attention in models in cognitive science outside of the foraging literature. So this co-occurrence deserves to be highlighted so that cognitive models that produce this co-occurrence can be developed.

We think it is an intriguing hypothesis that 1/f noise is not just a hallmark of complexity, but may also have functional purpose in cognition. While this is speculative, support for this hypothesis comes from Gilden, Thornton, & Mallon (1995) who found that 1/f noise disappears if the sequence becomes entirely unpredictable (and where cognition has no use). Similarly, as shown in the foraging literature, heavy tails in changes are adaptive when patchy environments are being explored (Viswanathan, Buldyrev, Havlin, Luz, Raposo, & Stanley, 1999, Nature). Our work points out that the co-occurrence of 1/f noise and heavy tails in cognition could have a functional interpretation in terms of a cognitive mechanism based on sampling. This of course does not imply that any co-occurrence of these statistics in a physical system indicates that cognition is occurring in that system.

My next concern pertains to evaluating whether MC^3 dynamics exhibit these properties. As far as I can tell, the authors fit lines in log-log space to either the power spectrum, or to the tail probabilities of difference distributions, and then adopted certain cutoffs for acceptable slopes. I find the logic of this approach a bit confusing, because one can always fit a line in log-log space. Spectra and tail probabilities will (nearly always) be diminishing, so the slope will be negative, and thus some exponent fitting the tail probabilities or power spectrum will be identified. So even fitting the tail of a normal distribution in this manner will yield a value of mu. The value of mu that will be obtained for non-power-law relationships will depend greatly on the range of values being considered. For instance, in fitting a power law to the tail of a standard normal distribution, I can get an exponent of -2 when considering z-scores of 1 to 2 (when considering z scores from 1 to 5, I get an exponent of -7.5). So a lot of the action in calling these distributions "power law" amounts to details of how the range of the tail is chosen, and what exponent cutoffs are used. Given the somewhat vague criteria for calling some difference distribution heavy-tailed, or some power spectrum 1/f, the pithy summary in Table 1 leaves much to be desired. For instance:

We agree with the Reviewer that identifying and fitting power-law are an important step for our work and if not done appropriately, we may identify wrong distributions as power-law or the other way around. In fact, though we did not explain this well enough in our initial submission, we did not just fit a line in the log-log plot. Instead, for mu, we followed the sophisticated method proposed by Clauset, Shalizi, & Newman (2009) *SIAM Review*. In particular, the simulations produced by MC3 and the other algorithms were fitted by a power-law distribution with the exponent and the range of data as free parameters. The range was calculated as the part of the dataset with the lowest Kolmogorov-Smirnov (KS) statistic. This was implemented by repeatedly increasing the lower bound and decreasing the upper bound until the KS statistic failed to improve consecutively five times. The power-law exponent was then fitted to this subset of the data using maximum-likelihood estimation. Here is an example of fitting MC3 generated data for both the animal naming task and the time estimation task. For mu, the bounds are indicated by the extent of the fitting line.

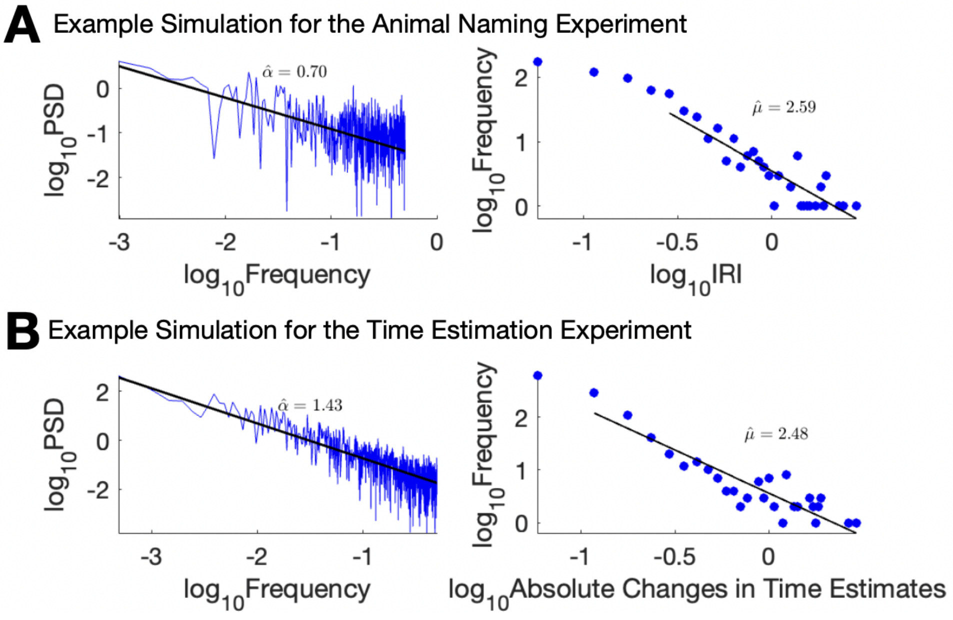

Figure 1. Example simulations of MC3 for the animal naming (A) and the time estimation (B) tasks. Within each panel from left to right, the plots are power spectrums for IRI (top) and time estimates (bottom) and histograms for IRI (top) and absolute changes in time estimates (bottom) in the log-log space.

The idea of exploring data generated from a Gaussian distribution is a good one, and we now include a "Validating the Data Fitting Methods" section in the Supplementary text that does this. We simulated i.i.d. samples from a Gaussian distribution of the same length that were generated from the other sampling algorithms, and fit both mu and alpha. Over 1,000 replications of this procedure, we found no replications that met either the heavy-tail criterion or the 1/f noise criterion.

- if one tried really hard in specifying a particular posterior distribution and proposal distributions, is it really the case that random walk metropolis and hamiltonian monte carlo would not be able to meet the criteria for alpha and mu? If some specifications regularly work, but some don't, what does that mean?

- on the flip side, is meeting the alpha and mu criteria roughly half the time really that compelling of evidence that MC^3 exhibits 1/f dynamics and heavy tailed change distributions?

Basically, both the negative, and positive conclusions drawn from Table 1 seem questionable to me, so I'm not sure how much we can conclude based on the differences among algorithms.

The analysis in our original submission established that for randomly selected parameters MC3 was more likely to produce heavy tails and 1/f noise than RWM or HMC. It would take some work to make RWM or HMC to meet the criteria for alpha and mu as often as MC3 does. The usual proposal distribution for RWM, for example, is a Gaussian, which clearly cannot generate heavy tails if the acceptance probabilities are high. However, one may think of an extended RWM whose proposal distribution is a power-law distribution. And if all the proposed samples were accepted from this power-law RWM, it will produce power-law in successive changes; but, at the same time, it will produce a random walk (i.e., 1/f^2 noise) instead of a 1/f noise (see response to Reviewer 3 regarding shuffled time series). Therefore, it requires an extensive and careful tuning of both proposal distribution and posterior distribution to mimic the co-occurrence of heavy tails and 1/f noise.

This careful tuning of RWM is difficult to motivate from a functional perspective. The efficiency of sampling algorithms decreases for higher autocorrelations, and so an algorithm with parameters tuned to produce 1/f noise instead of 1/f^2 noise (all else being equal) will be less efficient. MC3 is better motivated here because while it often shows 1/f scaling, it gains the ability to sample more easily from multi-modal probability distributions.

However, we take the point that our simulation does not make the case for MC3 on its own, as RWM and HMC do produce both heavy tails and 1/f noise for some parameterizations. What we need to establish is which algorithms fit the data best, and whether the best-fitting algorithm with its associated best-fitting parameters does reproduce the heavy tails and 1/f noise found empirically. We now have replaced that analysis with a Bayesian model comparison between MC3, RWM, and HMC and establish that MC3 is the best-fitting algorithm for time estimation and is competitive with the best algorithm (RWM) for animal naming. We also show that using the parameters that describe participants well, MC3 produces both heavy tails and 1/f noise.

Altogether, I am left with the impression that (a) the co-occurrence of heavy-tailed distributions and 1/f dynamics is not unique to cognition, so any cognition-specific explanation of the co-occurrence seems overly narrow, and (b) the tests that aimed to evaluate whether particular inference algorithms also exhibited these characteristics seemed not very diagnostic. The net result leaves much to be desired. Moving forward, I would like to see some stronger support for the claim that the conjunction of heavy tails and 1/f dynamics is somehow unique to cognition, and more rigorous evaluations of these properties in candidate algorithms.

We hope that we have addressed these concerns by 1) clarifying that we are exploring what the co-occurrence of heavy tails and 1/f noise in human behavioral data means for models of cognition, rather than proposing that the co-occurence is a general signature of cognition in any physical system, and 2) by showing that the fitting methods used to establish that MC3 produces the co-occurrence are diagnostic.

Reviewer #3: Review of “Understanding the Structure of Cognitive Noise”, submitted to PLOS Computational Biology (PCOMPBIOL-D-21-01984)

Reviewed by Peter M. Todd, Indiana University Cognitive Science Program

This is a compelling paper that convincingly shows that two types of noise seen in human behavior can systematically co-occur, and demonstrates a cognitive sampling mechanism that can produce that systematicity. The two empirical tasks are appropriate for making these points, along with the comparison between three sampling algorithms to fit the human data. The authors give support for the useful perspective that noise can be “the signature of a rational approximation in action” rather than an indication of cognitive or neural error as others have been arguing. Overall, the paper makes an important contribution on an interesting question with theoretical impact, is well written, and contains sufficient detail to afford replication and further research.

We thank the Reviewer for these kind comments.

It is good to see two quite different tasks—naming animals and estimating durations—being used to show instances of co-occurrence of the two noise patterns under consideration. Then for the possible underlying mechanism as a sampler over a particular task-related distribution, the applicability of a multimodal distribution to the animal naming task is clear, as people produce multiple clusters of related animal names, but how does it apply to the time estimation task? Is there the expectation in the time literature that an individual would have multiple likely time estimate values (multiple modes in the distribution) that they would be sampling from (rather than one mode)? Or is the point to show that in a task that probably has a unimodal outcome distribution, the Metropolis-coupled MCMC algorithm can still (at least sometimes) show both heavy-tailed and 1/f noise patterns? This should be clarified.

We agree with the Reviewer that the time representation is likely unimodal, as the distributions of estimates participants produce look unimodal. We think it is plausible however that if distributions are often multimodal, then MC3 would still be used on those occasions when the distribution is unimodal. One reason we might expect this is that MC3 only has access to local information of the target distribution (at multiple points, which is not enough to assess the global shape of the distribution), and so it would be very difficult for the sampling algorithm to be sure that there were not any far-away modes that were yet to be explored.

Indeed, MC3 does often still show both heavy-tailed changes and 1/f noise for unimodal distributions, as was the case in the simulations in our original submission and as we demonstrate here in the results of our new model comparison. We have added discussion to the text to clarify this point.

The Metropolis-coupled MCMC algorithm used here switches between patches based on comparing the current patch with another one that has already been discovered, which could possibly be what is happening via parallel search in someone's mind, but seems unlikely for individual animals foraging among patches in space. Humans also appear to use some patch-switching rules analogous to those used by foraging animals, in some tasks involving mental search (e.g., a giving-up-time rule—see Wilke et al., 2009). It could be interesting to see how this algorithm could work with such patch-switching rules that only use "local" information about the current patch, not about other available patches.

Wilke, A., Todd, P.M., and Hutchinson, J.M.C. (2009). Fishing for the right words: Decision rules for human foraging behavior in external and internal search tasks. Cognitive Science, 33, 497-529.

This question also points to a difference between the two types of tasks used here—in the standard animal naming task, there is the aspect of "using up" the resources in each visited mode, since animal names are supposed to not be repeated, and hence there is in some sense an increasing impetus driving the person to switch from the current mode/patch to a new one (or at least, there's a benefit to switching at some point—though perhaps not in this paper's particular version of the task, where repetitions *are* allowed), but in the time estimation task, that impetus does not seem to be present—why should a person (or the sampler) move away from a given mode in this case? (And again, as mentioned above, why expect that there *is* more than one mode in the time estimate distribution?)

Both the RWM and HMC algorithms use only local information in order to switch between patches, and as a result both algorithms will do so less effectively than MC3. However, RWM and HMC are certainly not the only algorithms that use local information and the foraging literature contains a wide variety of algorithms that could be evaluated here. That work is beyond the scope of our paper, particularly as in our tasks the resources are not "used up": participants are allowed to revisit old hypotheses.

However, we do now draw attention to this possibility, as well as to potential overlaps between foraging rules and sampling algorithms: any foraging rule that visits locations according to their probability is also a sampling algorithm.

Regarding the question as to why a person should move away from the mode in the time estimation task: without knowing the global structure of the distribution (e.g., its unimodality) they cannot be sure that the peak that they have reached is the absolute best response. Relatedly, if the distribution is multimodal it can be faster to sample rather than to optimize (Ma, et al., 2019).

Reference:

Ma, Y. A., Chen, Y., Jin, C., Flammarion, N., & Jordan, M. I. (2019). Sampling can be faster than optimization. *Proceedings of the National Academy of Sciences*, *116*(42), 20881-20885.

Finally, as another way to think about the likelihood of the co-occurrence of heavy tails and 1/f noise, given a particular set of values that are drawn from a heavy-tailed distribution, how likely is any particular sequential ordering of those values to show long-range autocorrelations like 1/f noise? (For example, what is the proportion of scrambled re-orderings of the data from these two tasks that still show 1/f autocorrelation?) Does this differ if the values are produced by a Lévy distribution versus by a non-blind patchy foraging process like area-restricted search operating on particular kinds of patch-size distributions (e.g., the latter could produce long IRIs/durations that are more regularly spaced across a sequence than the former)? If so, could this distinguish the processes going on in the two paradigms here (memory search and time estimation), possibly indicating why all participants showed both patterns in memory search but about half of participants showed both patterns in time estimation?

To test this, we repeatedly reshuffled the time series for both the animal naming task and the time estimation task (1000 reshuffles). For the animal naming task, the reshuffled time series, across repetitions, had a 0.06% chance of being classified as 1/f noise. For the time estimation task, the reshuffled time series, across repetitions, had

essentially a 0% chance of being classified as 1/f noise. These values are both very low, and argue against 1/f noise occurring by chance for values independently drawn from a heavy-tailed distribution. In addition, in response to R2, we also looked at the chance of time series being classified as 1/f noise if they were generated independently from a Gaussian distribution, and we found that this also did not occur across 1,000 repetitions.

Detailed comments by page (using manuscript-page numbers):

MS Page 5 top: “Conversely, the most common model of heavy tails in successive changes, the Lévy flight, is a random-walk model that does not produce long-range autocorrelations [7].” –this is presumably because the Lévy flights are assumed to apply to blind foraging over patchy resources with no memory, in contrast to commonly-seen area-restricted search strategies that do involve memory (e.g. making small movements to stay foraging locally as long as resources are plentiful) and hence can produce autocorrelation.

Thanks, we now acknowledge that area-restricted search strategies with memory can produce autocorrelation and should be investigated in future work. One interesting topic to explore is whether they can produce the co-occurence of heavy tails and 1/f noise when there is a unimodal distribution (i.e., only one patch) as MC3 can do.

P5 bottom: “all individual exponents indicate heavy-tailed distributions”—indicate what criterion is used to make this assessment (e.g. some particular range of tail exponent mu?). (I assume the criterion for 1/f noise given at the bottom of p. 3 is what is being used for that classification in the paper.)

The criterion for heavy tails was mu falling within (1,3], which was the standard set by Rhodes and Turvey (2007). We now make this explicit in the text. Yes, the range of [0.5,1.5] was used as the criterion for 1/f noise.

P5 bottom: “participants were more likely to report sequential animal names that belonged to the same category (e.g., patch) than other categories”—replace “sequential” with “successive” to emphasize that this is about the movement from one animal name to the very next one (not necessarily a longer sequence)?

Updated.

Pp5-6: the heavy-tail exponent estimates (mu) should be given for both tasks here (also so they can be compared with the mu estimates provided in the Supplementary Text on pp. 16-17).

Added relevant information in the text.

P13 top: It is surprising to see that participants could resubmit the same animal name repeatedly, which is not typically allowed in this verbal fluency task (e.g., not in Rhodes & Turvey, who specified “without repetition” but still got around 4 repetitions over 20 minutes). Why was that done here? Perhaps to get around the potential problem of “using up” resources in this task, which differs from the time estimation task? (see comment above) How often *did* participants repeat animal names, and did they do so while still in a particular patch, or later after returning to a specific patch?

We did this mainly because we needed very long sequences in order to estimate the 1/f exponent. A second benefit was that it removed the need to de-trend IRIs (as Rhodes and Turvey did) because they become progressively longer as animal names are used up.

On average, 3.63% of animal names were repeated (minimal and maximal proportions of repetitions were 0.38% and 10.70% respectively).

P18 middle, Algorithm 1: the call to MCMC Step is missing the second argument (of three).

Thank you – we’ve added the second argument in the function

P19 middle, Algorithm 2: in the line “for s = 0 : M//2 do” M is undefined.

Specified M at the top of the algorithm as the number of parallel chains

P20 middle, Algorithm 3: Why are there even line numbers? In line (4), l is undefined (should it be 1?). And in line (5), “for l:L do”, shouldn’t this be “for j=1:L do” (l is undefined)?

Corrected the pseudocode.

P21 top: “We evaluated power-law exponents and sample autocorrelations for MCMC, MC3, and HMC” – should MCMC be RWM (as on the previous line)?

Changed MCMC to RWM where appropriate.

P22 middle: should “MCMC” everywhere it appears in the last paragraph be “RWM”? And why is MMMD used later in the paragraph when just M is used at the beginning—do these refer to two different values?

Changed M_{MMD} to just M as it should represent the mean MMD.
