# Supplementary material for: Understanding the structure of cognitive noise
Source: PLoS Comput Biol. 2022 Aug 17;18(8):e1010312. doi: 10.1371/journal.pcbi.1010312 (PMC9423631; doi:10.1371/journal.pcbi.1010312)
Supplement: Attachment — Submitted filename: r2_response_letter.pdf [file pcbi.1010312.s003.pdf]

Dear Dr. Gershman,

Thank you for your very encouraging letter and for the opportunity to revise and resubmit our manuscript. In our revision, we have addressed the remaining comments of Reviewer 3 by adding and modifying text in the Introduction, Results, Discussion and Conclusions, and the Supporting Information. We provide replies to the individual comments in blue text below.

We hope you feel that this version is ready for publication, and we'd like to thank you and the reviewers for all your input on the paper, which has strengthened it significantly.

Best wishes,

Zhu, Pablo, Nick, and Adam

Dear Dr. Zhu,

Thank you very much for submitting your manuscript "Understanding the Structure of Cognitive Noise" for consideration at PLOS Computational Biology. As with all papers reviewed by the journal, your manuscript was reviewed by members of the editorial board and by several independent reviewers. The reviewers appreciated the attention to an important topic. Based on the reviews, we are likely to accept this manuscript for publication, providing that you modify the manuscript according to the review recommendations.

Please prepare and submit your revised manuscript within 30 days. If you anticipate any delay, please let us know the expected resubmission date by replying to this email.

When you are ready to resubmit, please upload the following:

[1] A letter containing a detailed list of your responses to all review comments, and a description of the changes you have made in the manuscript. Please note while forming your response, if your article is accepted, you may have the opportunity to make the peer review history publicly available. The record will include editor decision letters (with reviews) and your responses to reviewer comments. If eligible, we will contact you to opt in or out

[2] Two versions of the revised manuscript: one with either highlights or tracked changes denoting where the text has been changed; the other a clean version (uploaded as the manuscript file).

Important additional instructions are given below your reviewer comments.

Thank you again for your submission to our journal. We hope that our editorial process has been constructive so far, and we welcome your feedback at any time. Please don't hesitate to contact us if you have any questions or comments.

Sincerely,

Samuel J. Gershman

Deputy Editor

PLOS Computational Biology

****

A link appears below if there are any accompanying review attachments. If you believe any reviews to be missing, please contact ploscompbiol@plos.org immediately:

Reviewer's Responses to Questions

Comments to the Authors:

Reviewer #1: I appreciate the authors' thoughtful responses to my comments and the helpful changes they made to the paper. I have no further concerns and think the paper is ready for publication.

Great, thanks.

Reviewer #3: Review of revision of “Understanding the Structure of Cognitive Noise”, submitted to PLOS Computational Biology (PCOMPBIOL-D-21-01984-R1)

Reviewed by Peter M. Todd, Indiana University Cognitive Science Program

Overall, the authors have done a thorough and (to me) mostly convincing job of responding to the reviewers' comments. It is good to see their clarification of their aim to show both that heavy tails and 1/f noise co-occur in some cognitive activities, and that standard cognitive models do not produce this co-occurrence, but MC3 does; a brief summary of this aim would be good to include in the text of the paper as well. It was also very helpful to see that long-tailed distributions by themselves do not imply 1/f patterns—that might also be helpful for readers to know and so briefly mention in the paper.

We thank the Reviewer for the positive feedback. We now make the aim of our work and the absence of a link between heavy-tailed distributions and 1/f noise more explicit in Introduction as well as Discussion and Conclusions and the Supporting Information.

Detailed comments by page:

P10 top: “In line with our experimental instructions, IRIs was on average proportional to the number of samples that were generated before the nearest animal name to the sampler's position changed.” – This further explanation of the relation between the sampling process and IRIs is very good to include, but it made me realize that I was unclear on another aspect of the sampling method, namely that samples are drawn from abstract points in the semantic space, *not* (solely) from points corresponding to particular words. This is clear for the time distribution in the first task, but perhaps should be filled in more for the semantic search task, e.g. that somehow the mind is sampling abstract semantic representations and only when these get close enough to the location of an actual word in the space is that word produced. It is also not clear to me in the above sentence what part of the instructions is meant here, and how the instructions would affect the relationship between (human-generated) IRIs and (model-generated) number of samples—clarify.

We thank the Reviewer for this comment, and have clarified the nature of the semantic space we are sampling from with, “The distribution we used (shown in Figure 2) was obtained by fitting a Gaussian mixture model to the animal names arranged in a two-dimensional abstract semantic space in which the sampled points could lie between animal names (see Appendix B for details).”

We have also expanded on what was meant by “in line with our experimental instructions” now stating, “In line with our experimental instructions that asked participants to report an animal name as soon as the animal name they had in mind changed”.

P10 top: “Further details about these assumptions and an exploration of an alternative in which IRIs are related to the distance between samples are given in Appendix A.” – this actually appears at the end of Appendix B, not A. It is good to see this comparison with an alternative cognitive model as a robustness check, however the comparison is somewhat concerning, because it shows that the results are *not* robust to this small change in the model, which makes HMC outperform MC3 and RWM (but not outperform MC3 and RWM in the original model in Table 1). This

should be discussed more, to help readers decide what the implications of this result are for the overall generalizability of the results and conclusions of the paper.

We thank the Reviewer for pointing out the index error, which is rectified. We have also added the sentence, “This of course means that conclusions about the sampling algorithms cannot be drawn without also specifying how the samples relate to behavior.” to footnote 2 in the main text.

P11 Discussion: “Across the two tasks, these results indicate that MC3 better described the human data compared to RWM and HMC.” – does this mean averaging/combining across the two tasks? This seems misleading, given that RWM (slightly) outperformed MC3 on animal naming.

We thank the Reviewer for the comment. The original description of model performance is indeed confusing. This sentence is now expanded to provide a clearer summary of the quantitative fit.

P12 Table 2: It is not clear to me what the “MC3 no” line in the table represents? It doesn’t seem to correspond to “human data” as mentioned in the table caption, which I expected. Clarify.

We now changed the column from “MC3” to “MC3 posterior predictives”.
